# EUAdb: A resource for COVID-19 test development and comparison

**DOI:** 10.1371/journal.pone.0255417

**Published:** 2021-08-04

**Authors:** Alyssa Woronik, Henry W. Shaffer, Karin Kiontke, Jon M. Laurent, Ronald Zambrano, Mariah Daley, Jef D. Boeke, David H. A. Fitch

**Affiliations:** 1 Department of Biology, New York University, New York, New York, United States of America; 2 Department of Biology, Sacred Heart University, Fairfield, Connecticut, United States of America; 3 Institute for Systems Genetics and Department of Biochemistry and Molecular Pharmacology, NYU Langone Health, New York, New York, United States of America; 4 Department of Ophthalmology, New York University School of Medicine, New York, New York, United States of America; University of Helsinki: Helsingin Yliopisto, FINLAND

## Abstract

Due to the sheer number of COVID-19 (coronavirus disease 2019) cases there is a need for increased world-wide SARS-CoV-2 testing capability that is both efficient and effective. Having open and easy access to detailed information about these tests, their sensitivity, the types of samples they use, etc. would be highly useful to ensure their reproducibility, to help clients compare and decide which tests would be best suited for their applications, and to avoid costs of reinventing similar or identical tests. Additionally, this resource would provide a means of comparing the many innovative diagnostic tools that are currently being developed in order to provide a foundation of technologies and methods for the rapid development and deployment of tests for future emerging diseases. Such a resource might thus help to avert the delays in testing and screening that was observed in the early stages of the pandemic and plausibly led to more COVID-19-related deaths than necessary. We aim to address these needs via a relational database containing standardized ontology and curated data about COVID-19 diagnostic tests that have been granted Emergency Use Authorizations (EUAs) by the FDA (US Food and Drug Administration). Simple queries of this actively growing database demonstrate considerable variation among these tests with respect to sensitivity (limits of detection, LoD), controls and targets used, criteria used for calling results, sample types, reagents and instruments, and quality and amount of information provided.

## Background

In response to the growing threat of the COVID-19 pandemic, the FDA (US Food and Drug Administration) began issuing Emergency Use Authorizations (EUAs) to laboratories and commercial manufacturers for the development and implementation of diagnostic tests in February, 2020 [1]. So far, the gold standard assay for SARS-CoV-2 detection is the RT-qPCR (real-time quantitative polymerase chain reaction) test [2]. However, the authorized RT-qPCR test protocols vary widely, not only in the reagents, controls, and instruments they use, but also in the SARS-CoV-2 genes they target, what results constitute a positive SARS-CoV-2 infection, and their limit of detection (LoD). The FDA has provided a web site that lists most of the tests that have been issued EUAs, along with links to the authorization letters and summary documents describing these tests [1]. However, it is very time-consuming and challenging to use this site to compare or replicate these tests for a variety of reasons. First, at least 12 of 18 tests for EUA submissions made prior to March 31, 2020, are not listed there. To our knowledge, no EUAs have been issued for these applications. Second, the data are not standardized and are only provided as longhand prose in the summary documents. Third, some details (e.g. primer sequences) are absent from several of the test descriptions. Fourth, for tests provided by commercial manufacturers, summary documents are completely missing. To address at least the first three issues, we have developed a database, EUAdb (EUAdb.org), that holds standardized information about EUA-issued tests and is focused on RT-qPCR diagnostic tests, or “high complexity molecular-based laboratory developed tests” [2]. By providing a standardized ontology and curated data in a relational architecture, we seek to facilitate comparability and reproducibility, with the ultimate goal of consistent, universal and high-quality testing nationwide. Here, we document the basics of the EUAdb data architecture and simple data queries. To our knowledge, this is the first database that catalogs COVID-19 diagnostic testing protocols. The source files can be provided to anyone who wants to modify the database for his/her own research purposes. We ask that the original source of the files be made clear and that the database not be used in its original or modified forms for commercial purposes.

## Construction and content

**Fig 1:**
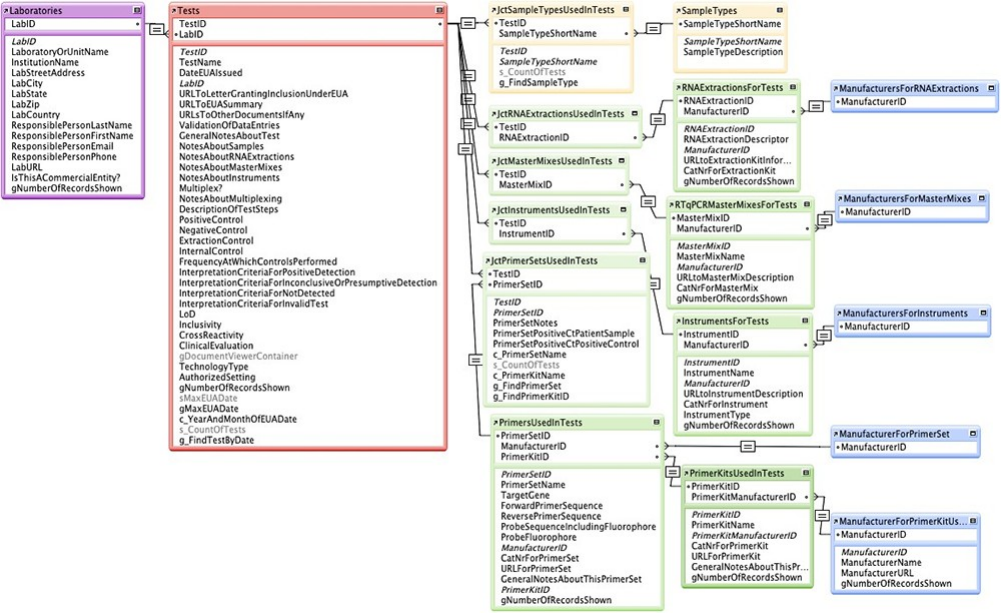
Main database architecture of EUAdb. Tables of data are related via one-to-many connections using a unique index field in each table, except for “join” tables that establish many- to-many connections via pairs of fields.

To construct EUAdb, FileMaker^™^ was chosen for its ease in building databases that can be freely shared online (e.g. via WebDirect^™^) without clients needing to use any tool other than a web browser. Although EUAdb is meant to be fairly straightforward and user-friendly for those with some knowledge of RT-qPCR tests, knowing a little about FileMaker will help users understand the data and generate custom queries. Fig 1 shows how the data from the FDA site is organized into tables and how these tables are related to each other. Laboratories that have applied for EUAs are represented with data sufficient to identify them, such as addresses, contact information, and URLs to the lab web sites. The Tests table includes data fields with URLs to documents stored on the FDA site, test-specific notes about sampling, reagents or instruments used, types of controls used, details about how results from the tests should be interpreted, etc. “Join” tables are used to allow many-to-many relationships between tests and sampling techniques, reagents or instruments. That is, because a test can use multiple sampling techniques, reagents or instruments, and the same sampling technique, reagent or instrument can be used in multiple tests, special tables with double index fields are needed to establish these relationships (tables with “Jct…” in their names). These join tables are then linked to tables for specific Sample Types, Nucleic Acid Extraction Kits, Master Mix Kits for PCRs, Instruments used for either nucleic acid extraction or RT-qPCR, and Primers that may be parts of Primer Kits used in the PCR. These tables are also linked to a Manufacturers table (represented by multiple occurrences, one for each reagent or instrument table). Fields, tables and architecture may change somewhat in the future as new tests are developed.

### Data curated and standardized ontology

In defining the specific data fields of the tables described above, we sought to standardize relevant data across all tests, thus allowing controlled and efficient data entry as well as comparisons among tests. However, the data provided for each test by the US FDA is in the form of a “summary document” written by the laboratory developing the test. Although the FDA requests certain types of information about each test, the summary documents are written in free-form prose and there is no easy way to automatically capture these data into individual fields. Thus, the information about each test is manually transferred from the summary document to the database by curators who are trained biologists and knowledgeable about RT- qPCR testing. This curation process required deciding on specific ontology, where the same reagent, instrument, etc. may be described with slightly different terminology in different summary documents. For example, we added some details that may not be explicitly found in the summary documents, such as catalog numbers, URLs to the item on the manufacturer web site and primer sequences (except in cases where the identity of specific reagents, instruments or manufacturers is unspecified or unclear). The data is entered by one person and independently validated by a second person.

## Utility and discussion

### Accessing and using EUAdb

**Fig 2:**
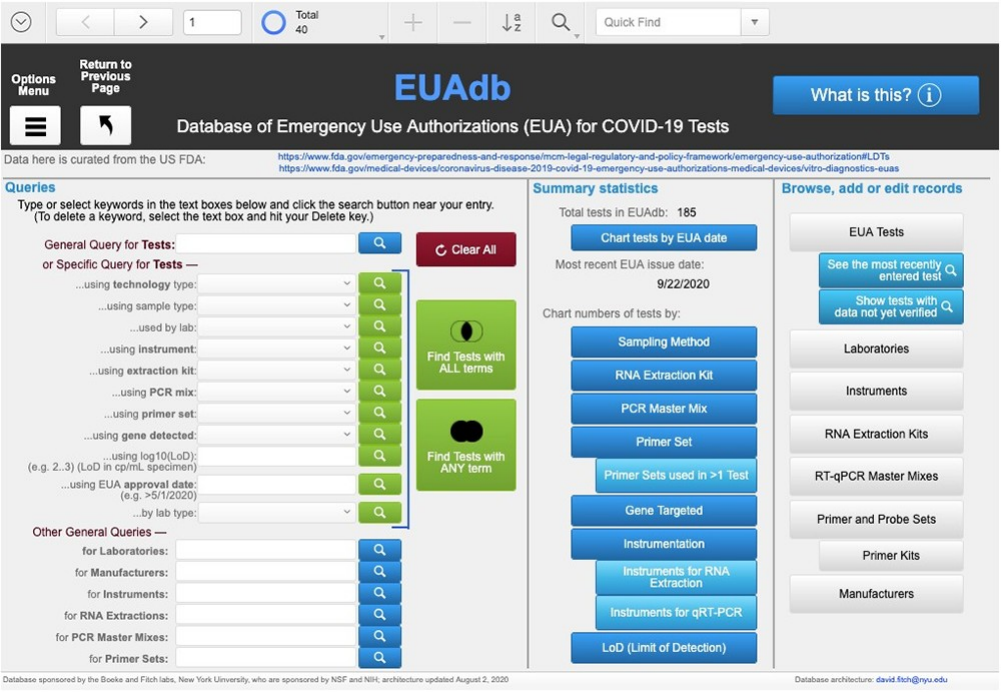
The Home layout is the landing page for EUAdb.org. See text for description.

Users can access all the data and perform all queries via an open Guest account at EUAdb.org. This URL leads to the Home page (Fig 2), which provides one-stop access to all data via global query fields and buttons attached to various scripts. In the upper left, Options- Menu and Return-to-Previous-Page buttons provide functions that are universally available on all layout pages. For example, clicking on the Options-Menu button pulls down a menu that allows you to choose the option to see a list of all tests or to exit the database. It is important to use the “Return to Previous Page” button instead of the browser “back” button because the database is run within a single browser window. Users are automatically signed out after 15 minutes of inactivity.

Several types of *Queries* are available by typing keyword(s) into field(s). Keyword fields next to blue query buttons allow any search term to be entered; clicking on the blue button will open a new page listing items that have the search term somewhere in the record. Several such free-form query fields are provided to query tests, labs, manufacturers, instruments and the different types of reagents. Keyword fields next to green buttons only allow particular terms to be selected in conjunction with a search for tests containing those terms. This allows a more controlled method of querying. For example, one can find all tests that use “Bronchial washes (BW)” as a sample type by selecting that term in the “Specific Query for Tests…using sample type” field and clicking the adjacent green query button. This action will bring up a page listing all tests in which bronchial washes are used as a sample type. More complex queries for tests can also be generated by selecting query terms for multiple kinds of items and using one of the large green buttons to find all tests consistent with “all” query terms (i.e., the Boolean intersection of sets of tests having each term) or with “any” of the query terms (i.e., the Boolean union of such sets). The red “Clear all” button provides an easy way to reset all terms to null values.

**Fig 3:**
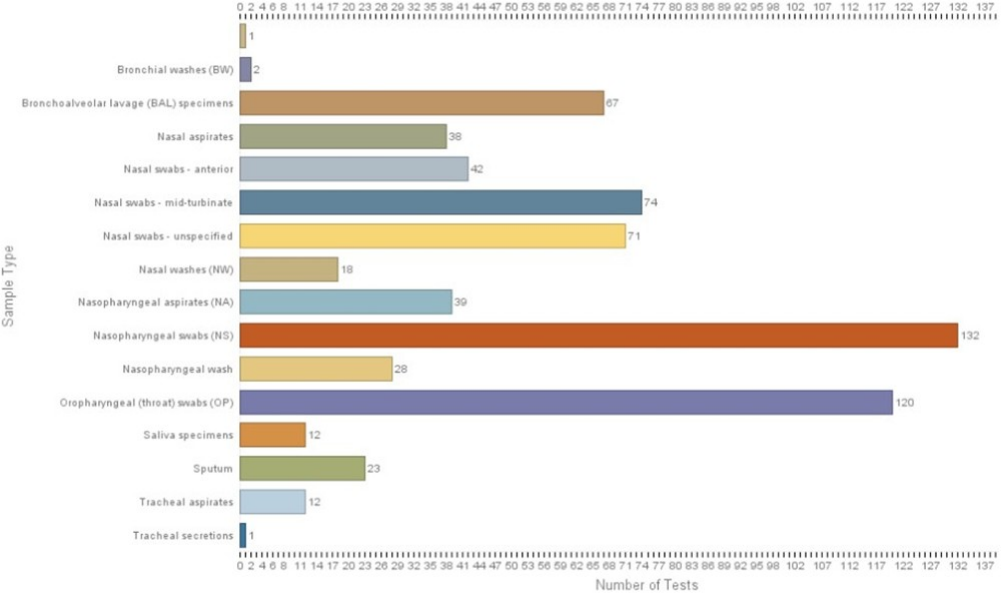
Example of a chart showing summary statistics for EUAdb, in this case the number of tests using a particular sample type. In the upper right corner is a query field for showing all tests that use a selected sample type. Such charts can also be generated for RNA extraction kits, PCR master mixes, primer sets, target genes, instrumentation, and limits of detection.

Several types of summary statistics are provided, including the number of tests recorded in the database, the most recent EUA issue date, and several charts about the tests and what kinds of samples, reagents or instruments are used in the tests. For example, clicking on the “Sampling Method” button brings up a page with a bar chart showing the numbers of tests that use each type of sample (Fig 3). This chart shows that nasopharyngeal swabs remain the most common sample type. To determine which tests these are, use the query box at the top right of the chart page to select “nasopharyngeal swabs” (you may need to select the down arrow in the value list to see additional values) and click on the green query button.

On the right side of the Home page are a series of buttons that allow Guest users to browse all database records. For those with FileMaker (FM) experience, typical FM functions are preserved at the top of the screen, allowing users a broader range of record navigation, query and sort functionality than provided on the Home page.

### A typical test page

**Fig 4:**
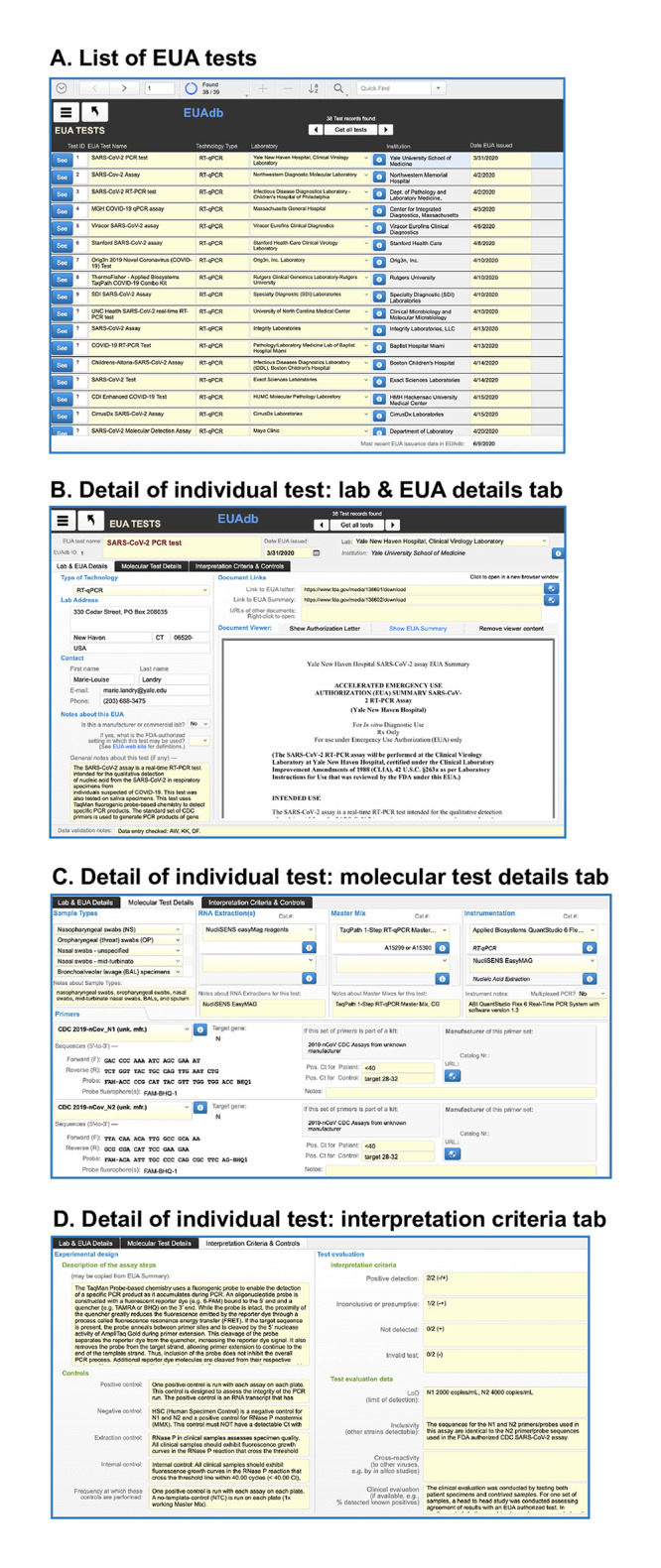
Example showing details for a particular test chosen from a found list of tests. See text for explanation.

Selecting “Browse all tests” from the Options menu will open a page listing all EUA tests entered into the database (Fig 4A). This list provides only a few fields of information about each test. To see all the curated details for any particular test, click the blue “See” button for that test in the list in order to open the Test page (Fig 4B). The main part of the Test page is a three-tabbed panel. The first tab is open by default and shows data about the laboratory and URLs for the FDA documents (Fig 4B). The original EUA Summary and other documents are available for viewing either within the document viewer, e.g. by clicking on “Show EUA Summary”, or by opening a separate browser window outside of the database, e.g. by clicking on the adjacent blue world-wide web buttons.

The second tab (opened by clicking on the “Molecular Test Details” tab title, Fig 4C) shows the sample types, nucleic acid extraction and PCR master mix reagents, instrumentation and primers used for a particular test. These data are shown as rows in “portals” linked to the join tables for those items. Additional information specific to an item may be obtained by clicking on an adjacent blue “i” button.

The third tab (Fig 4D) contains information about experimental controls and criteria for interpreting the test data. For example, the sensitivity of a test is reported as the LoD (limit of detection); notes about specificity are sometimes included as well.

Keyword-based queries for tests typically use the list of tests layout to show all the test records in the found set. However, if there is only one such record, the layout automatically switches to the test details layout.

### A motivating example of an EUAdb query

The motivation for making EUAdb.org was that NYU Langone was attempting to develop a COVID-19 PCR test protocol, thus in our illustrative example let’s assume there is a laboratory that wants to begin conducting COVID-19 PCR testing. The laboratory could begin by querying the database for the instrumentation or extraction kit they wished to use (i.e. what is available in their facility). They could then select the test with the greatest sensitivity for detecting SARS-CoV-2. For example, if a laboratory had an Applied Biosystems 7500 Fast Real Time PCR system and wanted to use the QIAamp Viral RNA Mini Kit for nucleic acid extraction, they could conduct an inclusive query of the database and discover that six tests with these criteria already have EUA approval. The laboratory would also be able to quickly determine that the limit of detection (i.e. the ability of the test to detect SARS-CoV-2) for these 6 tests ranges from 150 SARS-CoV-2 genome copies/ mL to 10,000 genome copies/ mL (determined by our Log10 LoD value). The laboratory would likely want to use the protocol that has the highest sensitivity (i.e. lowest Log10 LoD value) and could then also see what biological samples can be used as starting material for this test, the SARS-CoV-2 primers used, the Ct scores used to determine a positive test, etc. This ability to query tests saves the laboratory from needing to read through all the free-form prose documents that are posted to the FDA website for tests that utilize the instrumentation available in their facility.

### Findings using example queries

Queries of EUAdb reveal several interesting aspects about the diversity of the tests. A plurality—but not a majority—of the tests use the primers originally recommended by the CDC (US Centers for Disease Control and Prevention). The CDC primers target the *N1*, *N2*, and *N3* genes. Other SARS-CoV-2 regions that are targeted include *orf-1ab* and the genes encoding the E, RdRP, and S proteins. Some of the tests only use a single primer pair to detect viral genes, whereas most use at least two and sometimes three. The RT-qPCR result needed to declare a positive result for COVID-19 infection varies. For example, some tests with three primer pairs only require one amplification reaction to declare positivity, while others require at least two out of the three. The controls and sampling methods used also vary widely. There is no universally used sample method, although the nasopharyngeal swab is used in the majority of tests. Collecting nasopharyngeal samples is invasive and uncomfortable for the patient and might put health care workers at a higher risk of disease transmission [3]. These facts, in addition to swab and personal protective equipment shortages, have led to calls for the development of non-invasive, self-sampling methods using saliva [3]. As of this writing, only 13 tests can utilize saliva specimens (Fig 1) and an additional test can use oral rinses. At least 5 of these tests do not require the supervision of a healthcare worker during collection of the sample. The reagents used for RNA extraction and RT-qPCR vary substantially, as does instrumentation (although a plurality of tests use Applied Biosystems QuantStudio for qPCR). As recognized previously [4], the LOD (limit of detection) varies by several orders of magnitude across tests. Finally, we found that information provided by the FDA is incomplete with regard to several EUA applications announced prior to March 31, 2020. Of these, six are listed in separate tables on what appears to be a semi-redundant FDA web site [5]. We conclude that at least 12 pre-March 31 EUA applications are not listed on the FDA’s web site; we are contacting the owners of these EUAs to encourage them to provide their EUA submission materials for databasing in EUAdb.

## Conclusion

By providing standardized data and controlled ontology, EUAdb provides a useful and user-friendly resource for browsing, querying and comparing the different kinds of COVID-19 tests for which the FDA has issued EUAs [2]. Currently conducting such searches and comparisons on the FDA website are time consuming and difficult. In the future, maintaining such information in a more easily searchable/comparable format would likely be beneficial to rapid test development. Looking forward, the database could be used to see which technologies (including information technologies) might be best applied in future epidemics/pandemics to avoid replicated efforts and cryptic information and to promote the most effective diagnostics and information transparency.
